# Effects of pressure on morphology and structure of bio-char from pressurized entrained-flow pyrolysis of microalgae

**DOI:** 10.1016/j.dib.2018.03.048

**Published:** 2018-03-16

**Authors:** Kristina Maliutina, Arash Tahmasebi, Jianglong Yu

**Affiliations:** a Key Laboratory of Advanced Coal and Coking Technology of Liaoning Province, School of Chemical Engineering, University of Science and Technology Liaoning, Anshan 114051, China; b Chemical Engineering, University of Newcastle, Callaghan, NSW 2308, Australia

**Keywords:** Microalgae, Bio-char, Particle swelling, Pressurized pyrolysis

## Abstract

The present dataset describes the entrained-flow pyrolysis of Microalgae *Chlorella vulgaris* and the results obtained during bio-char characterization. The dataset includes a brief explanation of the experimental procedure, experimental conditions and the influence of pyrolysis conditions on bio-chars morphology and carbon structure. The data show an increase in sphericity and surface smoothness of bio-chars at higher pressures and temperatures. Data confirmed that the swelling ratio of bio-chars increased with pressure up to 2.0 MPa. Consequently, changes in carbon structure of bio-chars were investigated using Raman spectroscopy. The data showed the increase in carbon order of chars at elevated pressures. Changes in the chemical structure of bio-char as a function of pyrolysis conditions were investigated using FTIR analysis.

**Specifications Table**

**Table:** 

Subject area	Chemical Engineering, Carbon Materials
More specific subject area	Biomass utilization; Pyrolysis
Type of data	Table and Figure
How data was acquired	Pressurized entrained-flow pyrolysis, SEM-image analysis, Elemental Analysis, Raman spectroscopy, FTIR spectroscopy
Data format	Raw original data was collected and analyzed
Experimental factors	Pyrolysis experimental factors including pressure and temperature
Experimental features	Triplicate experiment. Prior to each trial, collected data was averaged and calculated to ensure repeatability
Data source location	Liaoning province, China
Data accessibility	All data are included in this document
Related research article	Maliutina, K., Tahmasebi, A., Yu, J., Saltykov, S.N. 2017. Comparative study on flash pyrolysis characteristics of microalgal and lignocellulosic biomass in entrained-flow reactor. *Energy Conversion and Management*, 151, 426–438. [1]

**Value of the Data**

• Data compares the results obtained under atmospheric and pressurized fast entrained-flow conditions.
• Data illustrated innovative information on changes in carbon structure and surface morphology of bio-chars.
• Data provided valuable information on changes in chemical structure of bio-chars as a function of pyrolysis conditions.
• The dataset is valuable for the development of efficient and innovative technologies for the production of biomass-based carbon materials.

## 1 Data

**Fig. 1:**
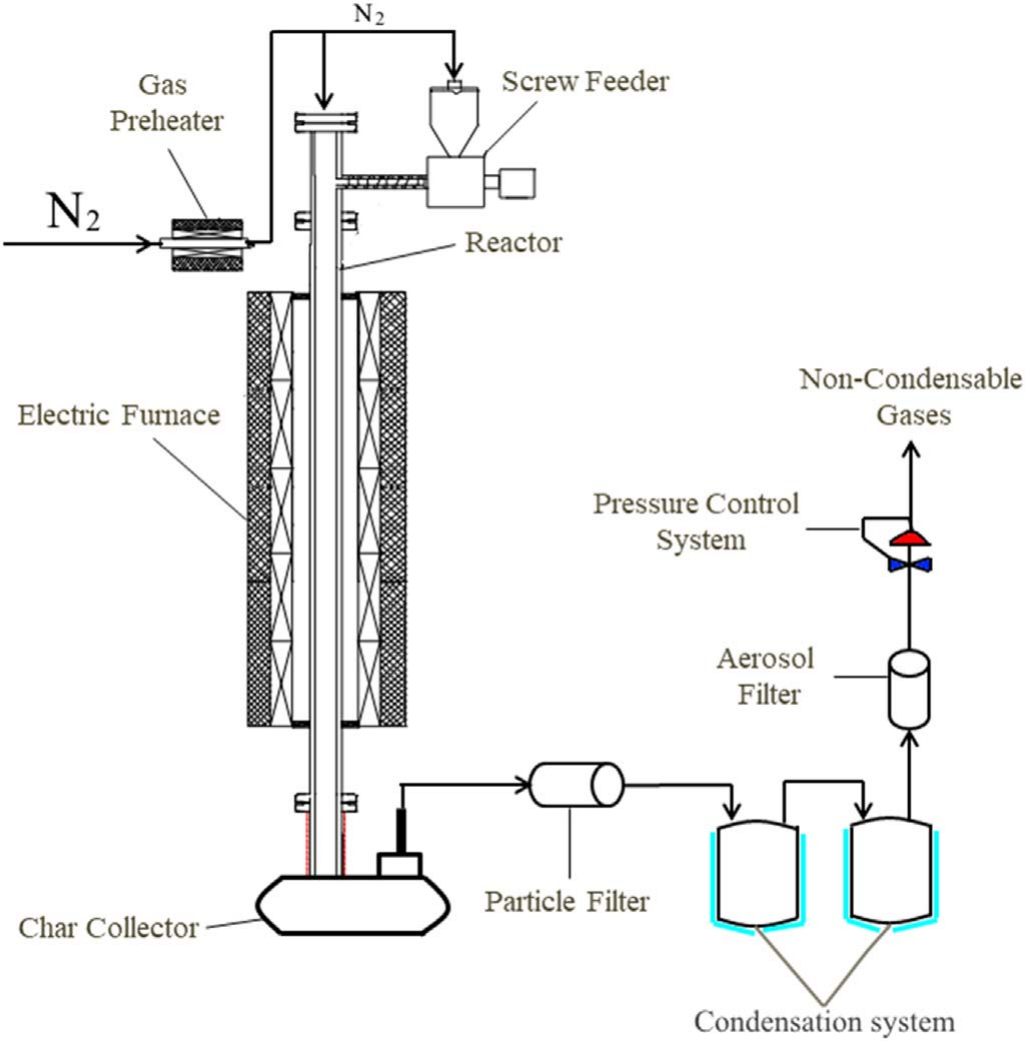
Schematic diagram of the pressurized entrained-flow pyrolysis experimental setup.

**Fig. 2:**
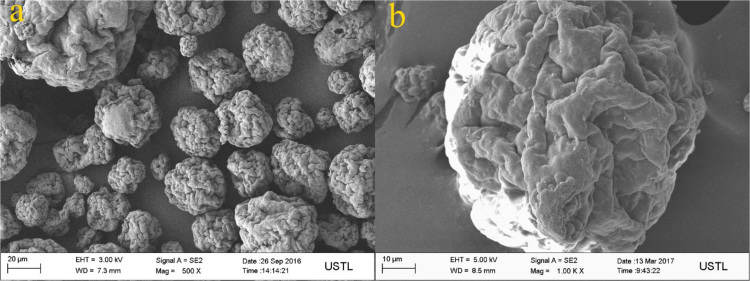
Scanning electron microscopy (SEM) images of raw Microalgae, where a and b shows different magnifications of the same sample.

**Fig. 3:**
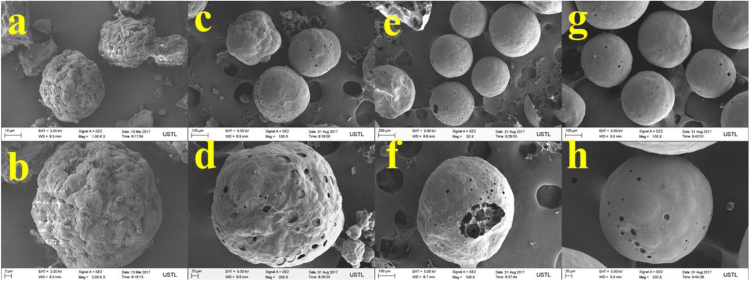
Scanning electron microscopy (SEM) images of bio-chars prepared at 600 °C and pressures of: (a, b) 0.1 MPa; (c, d) 1.0 MPa; (e, f) 2.0 MPa, (g, h) 4.0 MPa where a and b; c and d; e and f show different magnifications of the same sample.

**Fig. 4:**
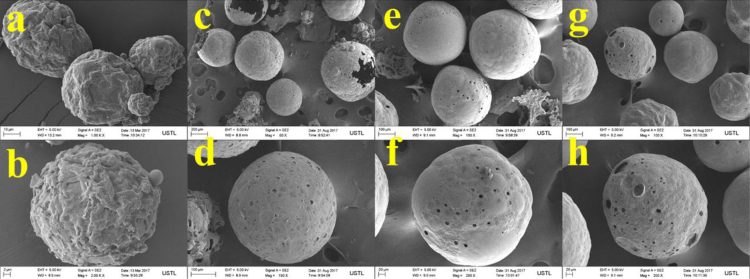
Scanning electron microscopy (SEM) images of bio-chars prepared at 700 °C and pressures of: (a, b) 0.1 MPa; (c, d) 1.0 MPa; (e, f) 2.0 MPa, (g, h) 4.0 MPa where a and b; c and d; e and f show different magnifications of the same sample.

**Fig. 5:**
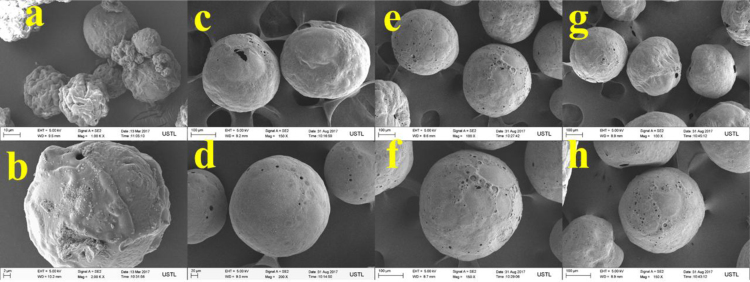
Scanning electron microscopy (SEM) images of bio-chars prepared at 800 °C and pressures of: (a, b) 0.1 MPa; (c, d) 1.0 MPa; (e, f) 2.0 MPa, (g, h) 4.0 MPa where a and b; c and d; e and f show different magnifications of the same sample.

**Fig. 6:**
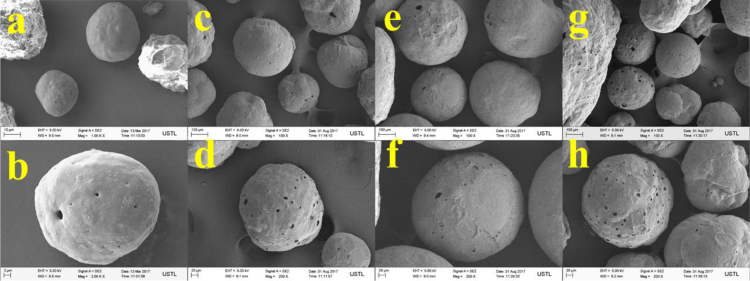
Scanning electron microscopy (SEM) images of bio-chars prepared at 900 °C and pressures of: (a, b) 0.1 MPa; (c, d) 1.0 MPa; (e, f) 2.0 MPa, (g, h) 4.0 MPa where a and b; c and d; e and f show different magnifications of the same sample.

**Fig. 7:**
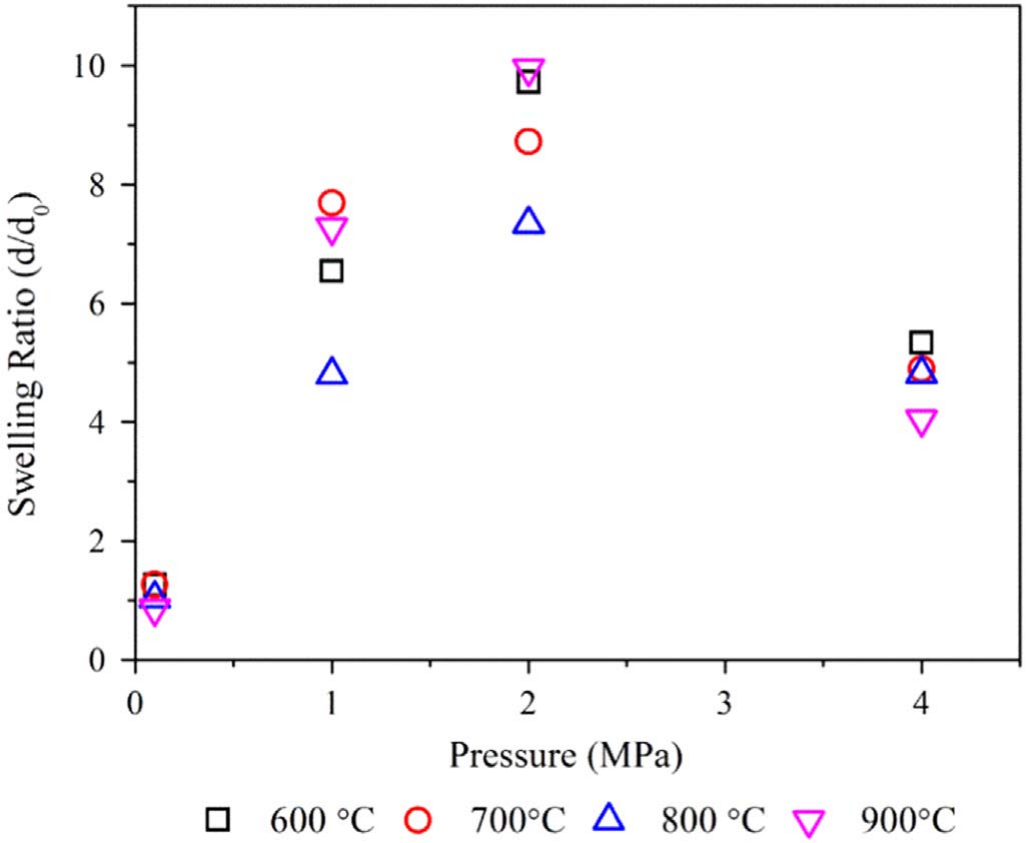
Swelling ratios of microalgae bio-chars as a functional of pyrolysis pressure.

**Fig. 8:**
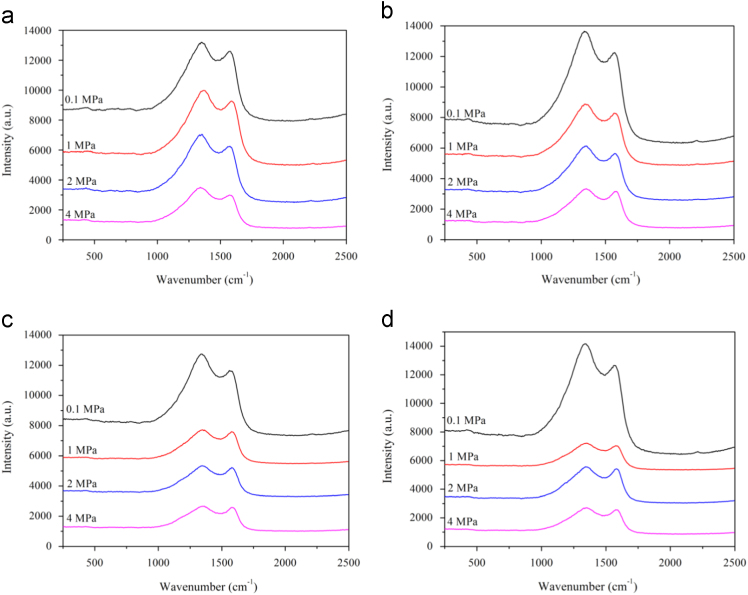
Raman spectra of microalgae bio-char samples: (a) 600 °C; (b) 700 °C; (c) 800 °C; and (d) 900 °C.

**Fig. 9:**
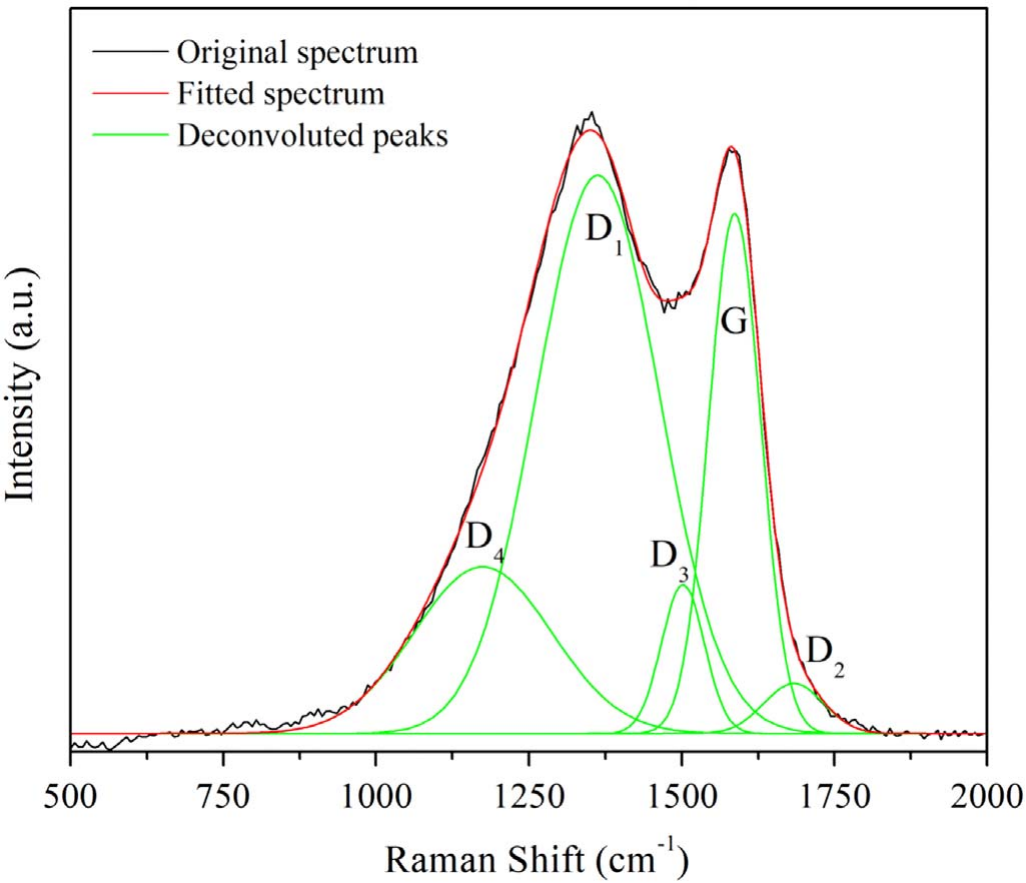
A typical curve-fitted Raman spectrum of bio-char.

The properties of raw microalgae biomass are presented in Table 1. Fig. 1 illustrates the schematic diagram of pressurized entrained-flow pyrolysis experimental setup. Fig. 2 and Fig. 3, Fig. 4, Fig. 5, Fig. 6 shows the scanning electron microscopy (SEM) images of microalgae and its bio-chars prepared under different temperatures and pressures at various magnifications, respectively. Swelling ratios of microalgae bio-chars under pressurized pyrolysis was measured from the SEM analysis data and are shown in Fig. 7. Fig. 8 shows the Raman spectra of microalgae and its bio-char samples prepared under different experimental conditions, while Fig. 9 shows the typical curve-fitted Raman spectra corresponding to D1, D2, D3, D4 and G1 bands, respectively. The FTIR curve-fitting analysis results of bio-chars are presented in Table 2.

**Table 1:** Characteristics of raw microalgae Chlorella vulgaris sample.

Sample	Elemental composition, (wt% daf)					Proteins, wt%	Carbohydrates, wt%	Lipids, wt%	M, (wt%, ar):,	VM, (wt%, db)	A, (wt%, db)	FC, (wt%, db)
	C	N	H	S	O							
Microalgae *Chlorella vulgaris*	43.85	7.86	6.07	0.71	41.61	56.44	17.3	12.4	10.21	80.41	5.54	14.05

**Table 2:** FTIR spectra curve-fitting analysis for raw microalgae and its chars prepared under different experimental conditions (area%).

Sample	Primary and secondary amines/amides	O-H	C-H alif	C=O	C-C arom	C-N arom	C-O	C-N-C chain	N-Q-N
	3430–3385 and 1690–1630	3500–3300	2980–2840	1712–1674	1639–1563	1388–1288	1275–1150	1243–1201	1121–1106
Raw Microalgae	31.36	51.22	10.45	50.83	37.28	4.62	6.72	7.52	–
Char 600 °C, 0.1 MPa	30.73	34.35	12.11	42.17	34.32	1.25	18.17	1.58	7.32
Char 600 °C, 1 MPa	28.21	10.86	11.87	31.54	33.50	2.04	14.34	3.21	9.39
Char 600 °C, 4 MPa	16.29	12.94	3.34	15.11	9.48	7.28	16.66	1.89	6.88
Char 700 °C, 0.1 MPa	29.93	9.79	6.29	17.81	15.72	2.71	10.69	1.59	3.80
Char 700 °C, 1 MPa	17.79	7.83	0.66	5.00	8.24	6.61	15.93	7.96	14.43
Char 700 °C, 4 MPa	1.02	3.29	0.76	9.38	8.62	4.40	5.89	2.52	18.36
Char 800 °C, 0.1 MPa	2.44	6.41	2.19	20.13	17.63	6.10	7.26	–	6.40
Char 800 °C, 1 MPa	2.28	3.14	0.73	1.97	10.8	3.92	–	–	11.08
Char 800 ° C, 4 MPa	–	1.96	–	1.47	9.69	6.89	–	–	12.66
Char 900 °C, 0.1 MPa	–	5.73	1.95	1.25	5.97	13.18	–	–	13.16
Char 900 °C, 1 MPa	–	4.76	0.30	6.55	1.92	10.87	–	–	16.51
Char 900 °C, 4 MPa	–	2.81	0.65	3.94	2.27	7.84	–	–	22.67

## 2 Experimental design, materials and methods

### 2.1 Sample preparation

Fresh water Microalgae *Chlorella vulgaris* was purchased from Spirulina Bio-Engineering Co. Ltd., China. The average particle size of microalgal sample was around 45 μm. Samples were prepared by drying in the oven at 105 °C for 24 h. Then, biomass samples were stored in a desiccator to avoid the moisture reabsorption prior to the further proximate and ultimate analyses. Characterization of microalgae was performed following ASTM standards of D3173 for the moisture, D3174 for the ash, and D3175 for the volatile matter, and the fixed carbon content was calculated by difference. Collected data of sample characterization are presented in Table 1, where: M, VM, A, and FC refer to moisture, volatile matter, ash, and fixed carbon, respectively. The methods followed for calculating the protein, lipids, and carbohydrate contents in microalgae can be found elsewhere [1].

### 2.2 Pressurized entrained-flow pyrolysis experiments

Pressurized pyrolysis experiments were carried out at temperatures of 600, 700, 800, and 900 °C and pressures of 0.1, 1.0, 2.0 and 4.0 MPa. Fig. 1 illustrates the schematic diagram of the experimental rig.

The tubular entrained-flow reactor with an inner diameter of 20 mm and the total length of the heating zone of 1500 mm was heated in an electric furnace with five separate controlled heating zones. A pressure regulator controlled the pressure of the system by back pressure valve. The sample-feeding rate was set at 0.5 g/min. High purity nitrogen (99.999%) with a flow rate of 5 L/min was used as the carrier gas. Prior to each trial, the carrier gas was preheated to 350 °C. The average residence time of pyrolytic vapors inside reaction zone was around 6 s. For each experimental run, approximately 30 g of dried microalgae was loaded to the feeder. The duration of each pyrolysis run was 30 min, after which the system was depressurized and the reactor was cooled to room temperature. After the experimental rig was depressurized and cooled, the bio-chars were removed from the char collector at the bottom of the reactor.

### 2.3 Methods

#### 2.3.1 SEM characterization

The surface morphology of microalgae and its bio-chars prepared under different pyrolysis conditions was obtained using scanning electron microscopy analysis (SEM, ZEISS Sigma HD). Fig. 2 shows the SEM images of raw microalgae sample, while Fig. 3, Fig. 4, Fig. 5, Fig. 6 show SEM images of bio-chars prepared under different experimental conditions of temperature (600,700,800 and 900 °C) and pressure (0.1, 1.0, 2.0, and 4.0).

It can be seen in Fig. 2 that raw microalgae had irregular nonporous morphology with cavities on the surface, while bio-chars particles underwent swelling and softening during pyrolysis, leading to higher sphericity and hollow structure. Based on the SEM analysis results it can be seen that compared with the chars prepared at atmospheric pressures, the sphericity and surface smoothness of the bio-char particles increased with increasing of pyrolysis temperature and pressure. Promotion of bubble formation was evident under the surface of microalgae bio-chars at high pressures. Devolatilization process of microalgae under pressure led to the formation of larger char particles and bobble growth with hollow structure indicating softening of bio-chars at elevated pressures.

Fig. 7 shows the swelling ratios of bio-char particles under atmospheric and pressurized pyrolysis conditions. The swelling ratios were calculated by dividing the average particle size of bio-chars to that of raw microalgae (d/d_0_). Data showed the highest swelling ratio of bio-chars at 2 MPa.

#### 2.3.2 Changes in carbon order of bio-chars

A Horiba Jobin Yvon Xplora plus Raman spectrometer was used for obtaining data for analyzing the carbon structure of bio-chars prepared under atmospheric and pressurized conditions. The spectrometer was equipped with a 50× lens that focused the 532 nm Ar laser beam. Three random bio-char particles were selected for analysis prior to each sample in the range of 500–2500 cm^−1^. For a better understanding of the changes in the microcrystalline structure of chars, a commercially available data processing program (Origin Plus) was applied for curve-fitting analysis of Raman spectra.

Fig. 8 shows changes in G and D bands in the Raman spectra of bio-char samples with pyrolysis pressure and temperature. It was observed that the G band of Raman spectra increased with increasing of pyrolysis pressure from atmospheric to 4 MPa monotonically. This was accompanied by a decrease in the D band corresponding to the defects in carbon structure of bio-char.

For further understanding of changes in the microcrystalline structure of bio-chars, the Raman spectra were curve-fitted to a series of five bands Peak assignments were selected according to the literature [1]. The band at 1586 cm^−1^ (G band) was assigned to the E_2g_ symmetry in graphite layers [2]. The D1 band at 1362 cm^−1^ was interpreted as aromatic clusters having more than 6 rings [3]. The D2, D3, and D4 bands at around 1683, 1501, and 1174 cm^−1^ were assigned as disordered surface graphene layers, functional groups and small aromatic clusters, and aromatic ethers, respectively [3], [4]. The higher full width at half maximum (FWHM) of the defect bands (D2, D3, and D4) in Raman spectra suggested that the carbon structure of bio-chars was highly amorphous in nature. A typical curve-fitted Raman spectrum of bio-char is illustrated in Fig. 9.

#### 2.3.3 Changes in the chemical structure of bio-chars

FTIR analysis was carried out for investigating the changes in surface functionalities in raw MA and its chars prepared under different experimental conditions. The regions where major changes occurred were 3500–3300 cm^−1^ (OH), 2898–2840 cm^−1^ (C−H_alif_), 1712–1674 cm^−1^ (C=O), 1639–1563 cm^−1^ (C-C_arom_), and 1275−1150 cm^−1^ (C−O). The nitrogen-containing functionalities corresponded to 3430–3385 cm^−1^ and 1690–1630 cm^−1^ (primary and secondary amines/amides, respectively), 1388–1288 cm^−1^ (C-N_arom_ bond in pyridinic and pyrrolic rings), 1243–1201 cm^−1^ (C-N-C bond in chain structure), and 1121–1106 cm^−1^ (quaternary bond N-Q-N) according to the literature [5], [6], [7]. The results of FTIR curve-fitting analysis for the raw MA and its chars are summarized in Table 2. The curve-fitting analysis of FTIR spectra was performed for the two regions of 2750–3500 cm^−1^ and 980–1720 cm^−1^ with 10 peak positions. The results of the curve-fitting analysis for 3430–3385 cm^−1^ and 1690–1630 cm^−1^ regions (primary and secondary amines/amides, respectively) were summarized and presented together.

It can be seen in Table 2, that the concentration of major functional groups decreased at elevated pyrolysis pressures due to devolatilization during pyrolysis to form light gases. The decrease in the intensity of OH groups indicated the decomposition of phenolic and alcoholic functional groups, while the decrease in concentration of aliphatic C−H groups in 2898–2840 cm^−1^ region indicated that methyl groups were released from aromatic rings [8]. The intensity of C−O band in methoxy groups decreased with pyrolysis temperature, resulting in the release of CO_2_ [9]. The intensity of the band in 1639–1563 cm^−1^ region, corresponding to aromatic nature of char decreased at higher pyrolysis temperature, indicating a decrease in aromatic substitution and decomposition of heteroatom functionalities to form oxygen and nitrogen-containing gases [9], [10]. Peaks corresponding to amine/amide functional groups were disappeared at higher temperatures and pressures, while pyridinic, pyrrolic (C-N_arom_) and quaternary (N-Q-N) structures were retained in bio-chars.
