# Supplementary material for: Effects of pressure on morphology and structure of bio-char from pressurized entrained-flow pyrolysis of microalgae
Source: Data Brief. 2018 Mar 16;18:422–31. doi: 10.1016/j.dib.2018.03.048 (PMC5996292; doi:10.1016/j.dib.2018.03.048)
Supplement: Supplementary file 1 — Supplementary material. [file mmc1.docx]

**Conflict of interest**

Dear Editor

We do not have any conflict of interest in publishing the research work in the Data in Brief.

Yours sincerely,

Jianglong Yu

PhD, A/Prof, MIEAust

(On behalf of all authors)
